# Supplementary material for: S-nitrosothiol homeostasis maintained by ADH5 facilitates STING-dependent host defense against pathogens
Source: Nat Commun. 2024 Feb 26;15:1750. doi: 10.1038/s41467-024-46212-z (PMC10897454; doi:10.1038/s41467-024-46212-z)
Supplement: Supplementary file 1 — Supplementary Information [file 41467_2024_46212_MOESM1_ESM.pdf]

Supplementary Tables

Table 1. Sequences of PCR primers used in this study.

Name	Primer	Sequence
mIfnb	Forward	5’-ATGAGTGGTGGTTGCAGGC-3’
	Reverse	5’TGACCTTTCAAATGCAGTAGATTCA-3’
mCxcl10	Forward	5’-ATCATCCCTGCGAGCCTATCCT-3’
	Reverse	5’-GACCTTTTTTGGCTAAACGCTTTC-3’
mIsg15	Forward	5′-AGAAGCAGATTGCCCAGAAG-3′
	Reverse	5′-TGCGTCAGAAAGACCTCATAGA-3′
mIsg54	Forward	5′-CCTAAACAGTTACTCCACCTTCG-3′
	Reverse	5′-TTGCTGACCTCCTCCATTCT-3′
mIsg56	Forward	5′-TGCTGAGATGGACTGTGAGGAA-3′
	Reverse	5′-TCTTGGCGATAGGCTACGACTG-3′
mMx1	Forward	5′-ATGGATTCTGTGAATAATCTGTGCA-3′
	Reverse	5′-CTATGTCTCCAAACTGGGAAGGG-3′
hIFNB	Forward	5′-CAACAAGTGTCTCCTCCAAAT-3′
	Reverse	5′-TCTCCTCAGGGATGTCAAAG-3′
HSV-1 UL30	Forward	5′-CATCACCGACCCGGAGAGGGAC-3′
	Reverse	5′-GGGCCAGGCGCTTGTTGGTGTA-3′
HSV-1	Forward	5′-ACGACAGTGGCATAGGTTGG-3′
	Reverse	5′-CCGACATCACAAGGGACCTC-3′
mAdh5	Forward	5′-CGCCTTTGGAGGATGGAAGA-3′
	Reverse	5′-GGAGAGATTGCCGGTCACAA-3′

Supplementary figures

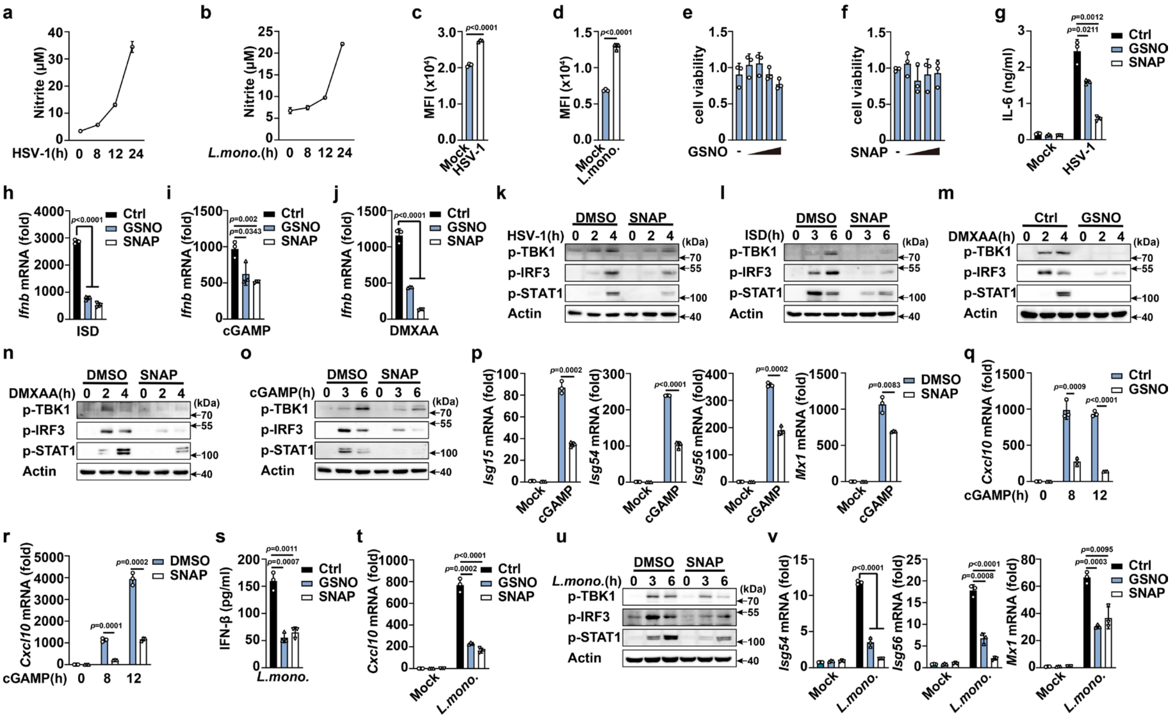

Supplementary Fig. 1: NO donors inhibit cGAS-STING activation. a,b, Nitrite analysis of supernatants of PMs were infected with HSV-1 (a) or *L. monocytogenes* (b). **c,d,** ROS analysis of PMs were infected with HSV-1 (c) or *L. monocytogenes* (d). **e,f,** Cell viability analysis of PMs treated with increasing concentrations of GSNO (0, 50, 100, 250, and 500 μM) or SNAP (0, 50, 100, 250, and 500 μM). **g,** ELISA analysis of IL-6 secretion in PMs pretreated with solvent (Ctrl), GSNO, or SNAP, and then infected with HSV-1. **h–j,** qPCR analysis of *Ifnb* expression in PMs pretreated with solvent (Ctrl), GSNO, or SNAP, plus stimulation as indicated. **k–o,** Immunoblot assays of p-TBK1, p-IRF3, and p-STAT1 in PMs pretreated with solvent (Ctrl), GSNO, or SNAP, plus stimulation as indicated. **p–r,t,** qPCR analysis of *Cxcl10*, *Isg15*, *Isg54*, *Isg56*, and *Mx1* mRNA expression in PMs pretreated with solvent (Ctrl), GSNO, or SNAP, and then stimulated as indicated. **s,** ELISA analysis of IFN-β secretion in *L. monocytogenes*-infected PMs pretreated with solvent (Ctrl), GSNO, or SNAP. **u,** Immunoblot assays of p-TBK1, p-IRF3, and p-STAT1 in *L.*

monocytogenes-infected PMs pretreated with DMSO or SNAP. **v,** qPCR analysis of *Isg54*, *Isg56*, and *Mx1* mRNA expression in *L. monocytogenes*-infected PMs pretreated with solvent (Ctrl), GSNO, or SNAP. Data represent mean ± SD or one representative from three independent experiments. The *p* values were calculated using unpaired two-sided t-test and adjustments were made for multiple comparisons.

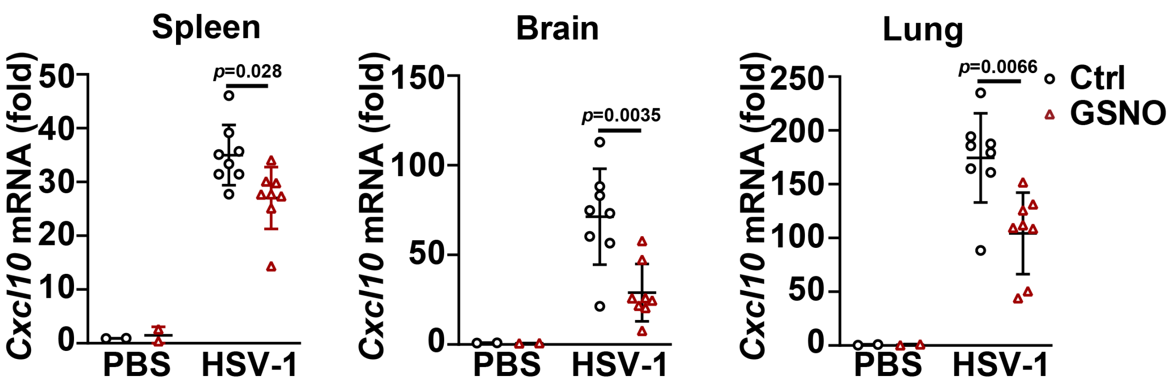

Supplementary Fig. 2: GSNO attenuates HSV-1 infection-induced *Cxcl10* expression *in vivo*. C57BL/6J mice were pretreated with solvent (Ctrl) or GSNO, and then infected with HSV-1 by intraperitoneal injection. qPCR analysis of *Cxcl10* mRNA expression in the spleen, brain, and lung tissues (PBS group, n = 2; HSV-1 group, n = 8). Data represent mean ± SD. The p values were calculated using unpaired two-sided t-test and adjustments were made for multiple comparisons.

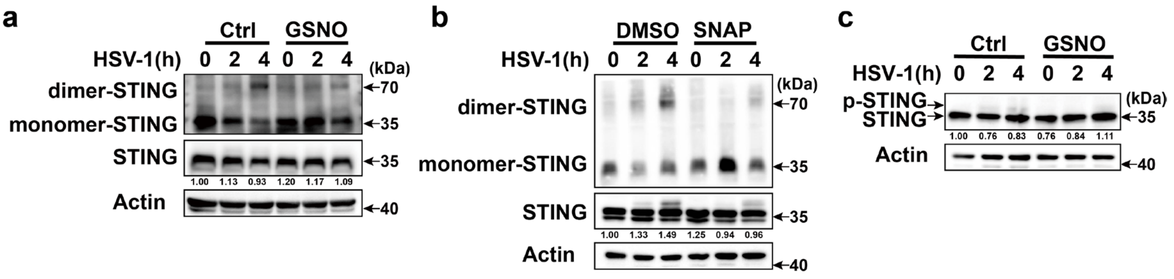

Supplementary Fig. 3: GSNO targets STING. a,b, Immunoblot assays of STING dimerization in PMs pretreated with solvent (Ctrl), GSNO, or SNAP, and infected with HSV-1. **c,** Immunoblot assays of STING and p-STING in PMs pretreated with solvent (Ctrl) or GSNO, and infected with HSV-1. Data indicate one representative from three independent experiments.

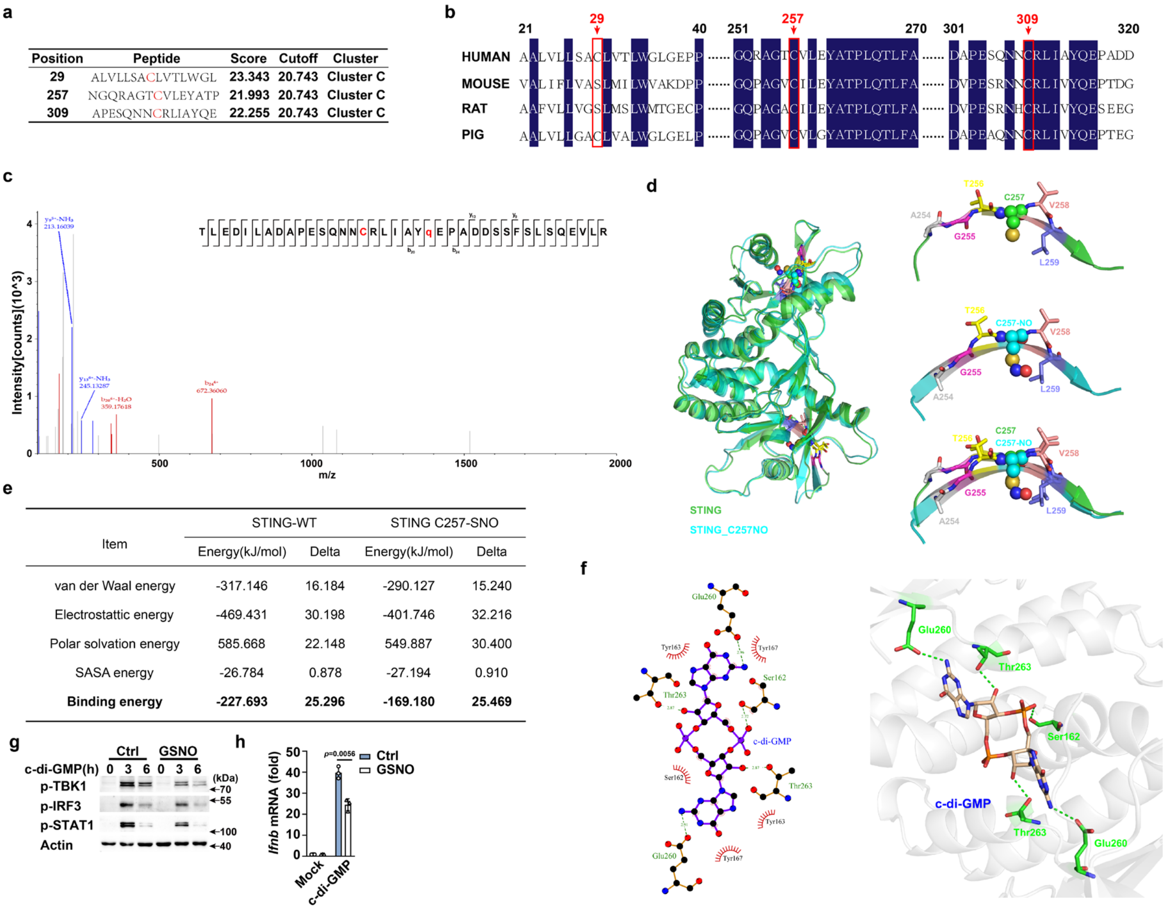

Supplementary Fig. 4: GSNO induces S-nitrosylation of STING to inhibit cGAMP binding. a, Potential S-nitrosylation sites of STING predicted by GPS-SNO 1.0. **b,** Conserved amino acid residues of STING in mammals. **c,** LC-MS spectra of STING at residue C309. **d,** Simulation of WT STING and S-nitrosylation of STING at C257 (STING-C257-NO) after 80–100 ns. **e,** Binding energy of cGAMP with STING-WT or STING-C257-NO after 80-100 ns simulation using MMPBSA calculates. **f,** Simulation of WT STING and S-nitrosylation of STING at C257 (STING-C257-NO) after 80–100 ns. **g,** Immunoblot assays of p-TBK1, p-IRF3, and p-STAT1 in c-di-GMP -stimulated PMs pretreated with solvent (Ctrl) or GSNO. **h,** qPCR analysis of *Ifnb* expression in PMs pretreated with solvent (Ctrl) or GSNO, plus stimulation as indicated. Data represent mean ± SD or one representative from three independent experiments. The *p* values were calculated using unpaired two-sided t-test and adjustments were made for multiple comparisons.

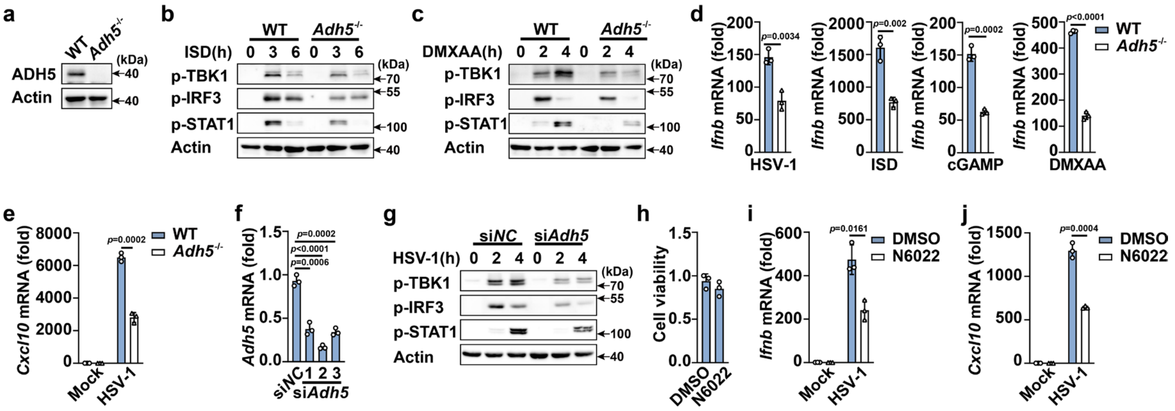

Supplementary Fig. 5: ADH5 facilitates STING activation. a–c, Immunoblot assays of ADH5, p-TBK1, p-IRF3, and p-STAT1 in PMs from *Adh5*$^{+/+}$ or *Adh5*$^{-/-}$ mice stimulated with ISD or DMXAA. **d,e**, qPCR analysis of *Ifnb* or *Cxcl10* mRNA expression in PMs from *Adh5*$^{+/+}$ or *Adh5*$^{-/-}$ mice, plus stimulation as indicated. **f,** qPCR analysis of *Adh5* mRNA expression in PMs transfected with negative control (si*NC*) or *Adh5* siRNA (si*Adh5-1,2,3*). **g,** Immunoblot assays of p-TBK1, p-IRF3, and p-STAT1 in PMs transfected with negative control si*NC* or si*Adh5-2*, followed by HSV-1 infection. **h,** Cell viability analysis of PMs treated with N6022. **i,j,** qPCR analysis of *Ifnb* or *Cxcl10* mRNA expression in PMs pretreated with DMSO or N6022 and then infected with HSV-1. Data represent mean ± SD or one representative from three independent experiments. The *p* values were calculated using unpaired two-sided t-test and adjustments were made for multiple comparisons.

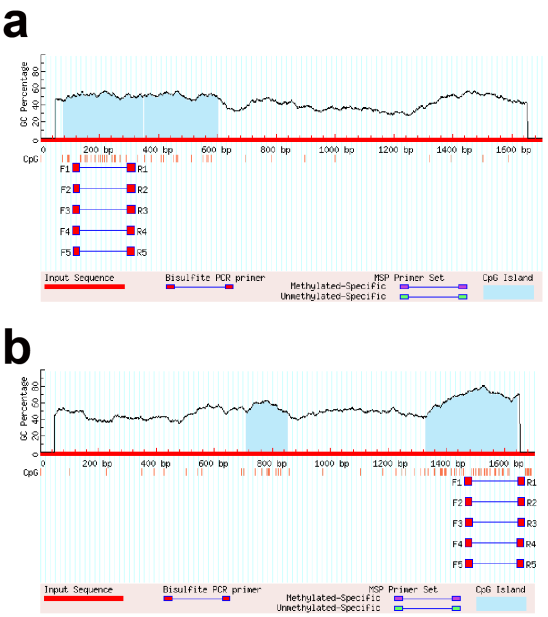

Supplementary Fig. 6: ADH5 is downregulated during pathogens infection by promoting DNA methylation. a,b, The promoter region of both human and mouse ADH5 revealed the presence of CpG islands.

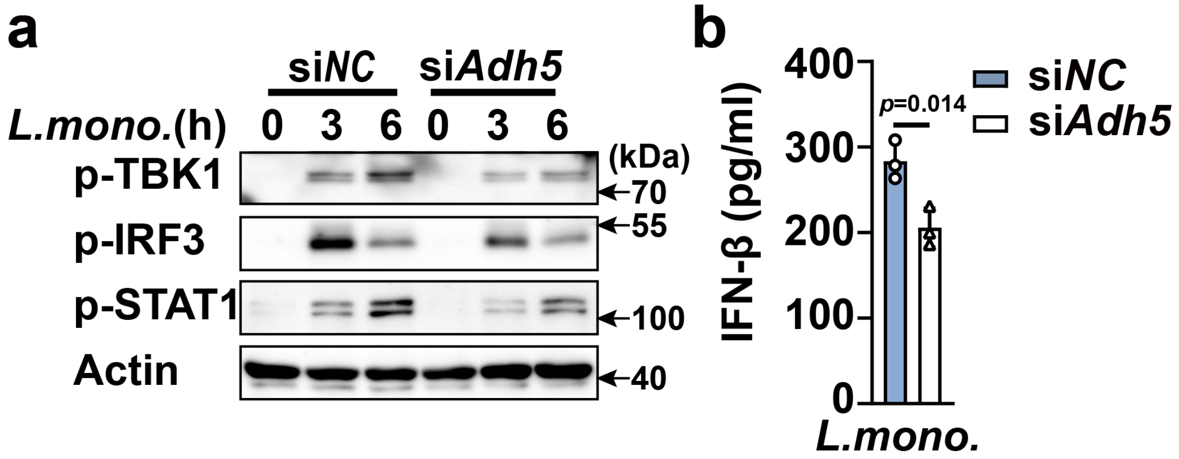

Supplementary Fig. 7: ADH5 attenuates *L. monocytogenes* infection-induced innate responses. **a,** Immunoblot assays of p-TBK1, p-IRF3, and p-STAT1 in PMs transfected with negative control (si*NC*) or *Adh5* siRNA (si*Adh5-2*), followed by *L. monocytogenes* infection. **b,** ELISA analysis of IFN-β secretion in *L. monocytogenes*-infected PMs transfected with negative control si*NC* or si*Adh5-2*. Data represent mean ± SD or one representative from three independent experiments. The *p* values were calculated using unpaired two-sided t-test and adjustments were made for multiple comparisons.

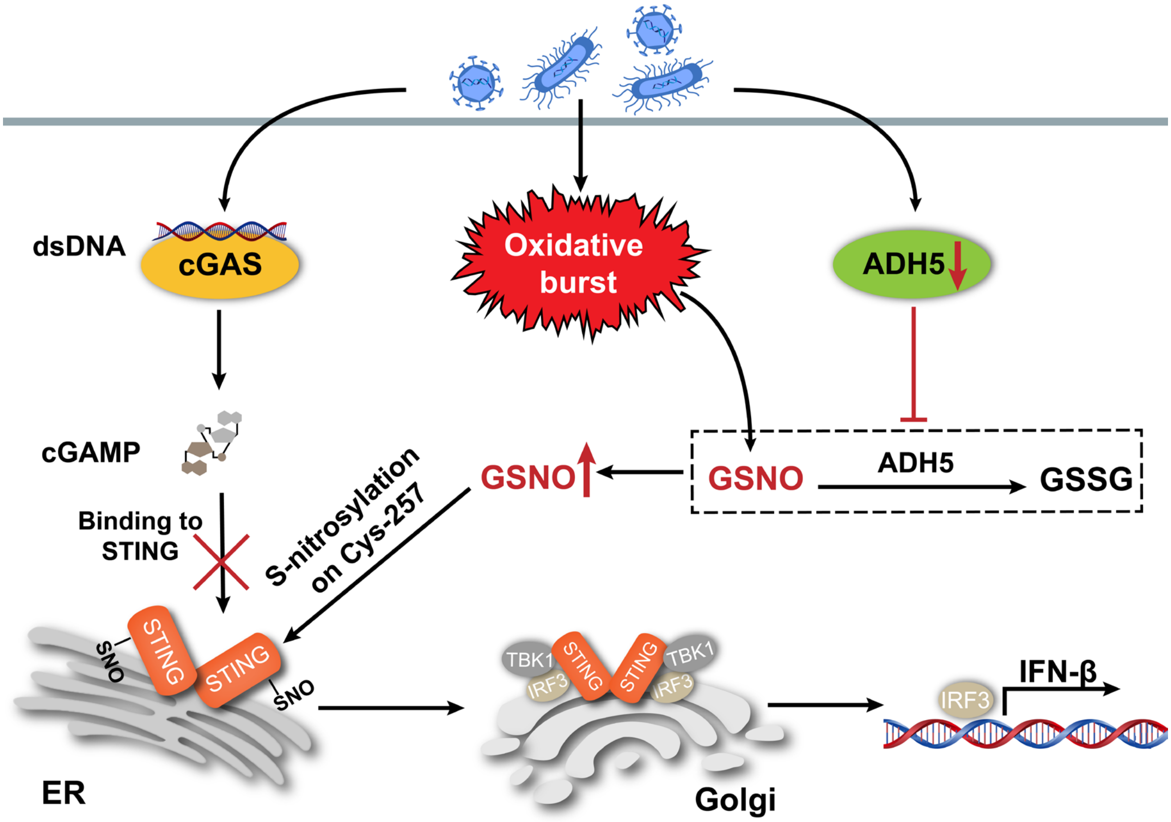

Supplementary Fig. 8: Schematic representation of the role of ADH5 in maintaining SNO homeostasis and controlling STING activity. ADH5, a critical enzyme that metabolizes GSNO to decrease total levels of SNO, activates STING to initiate the type I IFN response against pathogens. ADH5 is downregulated during pathogens infection to promote S-nitrosylation of STING and suppress its binding to cGAMP, thus circumventing host innate immune responses.
