# Supplementary material for: S-nitrosothiol homeostasis maintained by ADH5 facilitates STING-dependent host defense against pathogens
Source: Nat Commun. 2024 Feb 26;15:1750. doi: 10.1038/s41467-024-46212-z (PMC10897454; doi:10.1038/s41467-024-46212-z)
Supplement: Supplementary file 3 — Reporting Summary [file 41467_2024_46212_MOESM3_ESM.pdf]

nature portfolio

Corresponding author(s): Wei Zhao

Last updated by author(s): Feb 12, 2024

Reporting Summary

Nature Portfolio wishes to improve the reproducibility of the work that we publish. This form provides structure for consistency and transparency in reporting. For further information on Nature Portfolio policies, see our Editorial Policies and the Editorial Policy Checklist.

Statistics

For all statistical analyses, confirm that the following items are present in the figure legend, table legend, main text, or Methods section.

n/a	Confirmed	
☐	☒	The exact sample size (*n*) for each experimental group/condition, given as a discrete number and unit of measurement
☐	☒	A statement on whether measurements were taken from distinct samples or whether the same sample was measured repeatedly
☐	☒	The statistical test(s) used AND whether they are one- or two-sided<br>*Only common tests should be described solely by name; describe more complex techniques in the Methods section.*
☒	☐	A description of all covariates tested
☐	☒	A description of any assumptions or corrections, such as tests of normality and adjustment for multiple comparisons
☐	☒	A full description of the statistical parameters including central tendency (e.g. means) or other basic estimates (e.g. regression coefficient) AND variation (e.g. standard deviation) or associated estimates of uncertainty (e.g. confidence intervals)
☐	☒	For null hypothesis testing, the test statistic (e.g. *F*, *t*, *r*) with confidence intervals, effect sizes, degrees of freedom and *P* value noted<br>*Give P values as exact values whenever suitable.*
☒	☐	For Bayesian analysis, information on the choice of priors and Markov chain Monte Carlo settings
☒	☐	For hierarchical and complex designs, identification of the appropriate level for tests and full reporting of outcomes
☒	☐	Estimates of effect sizes (e.g. Cohen's *d*, Pearson's *r*), indicating how they were calculated

Our web collection on statistics for biologists contains articles on many of the points above.

Software and code

Policy information about availability of computer code

Data collection	CFX Connect Real-Time PCR Detection System (BIO-RAD,USA). Amersham Imager 680 (GE Healthcare Life Sciences,USA), Tanon 4800 (Tanon Science & Technology Co., Ltd., China), Infinite M200 Pro (Tecan,Switzerland), LSM 880 with Airyscan (Zeiss, Germany), Orbitrap Elite LC-MS/MS (Thermo, USA), Proteome Discoverer 1.4 (Thermo), Gromacs 2018.4, Cytoflex cytometer instrument (Beckman Coulter, Brea, CA, USA).
Data analysis	Prism 9.3(GraphPad,California,USA), ZEN blue and gray edition 3.1(Zeiss , Germany), Image J Version1.54f, FlowJo 10.8, The CpG islands of both human and mouse ADH5 were predicted on the website: http://www.urogene.org/cgi-bin/methprimer/methprimer.cgi.

For manuscripts utilizing custom algorithms or software that are central to the research but not yet described in published literature, software must be made available to editors and reviewers. We strongly encourage code deposition in a community repository (e.g. GitHub). See the Nature Portfolio guidelines for submitting code & software for further information.

Data

Policy information about availability of data

All manuscripts must include a data availability statement. This statement should provide the following information, where applicable:

- Accession codes, unique identifiers, or web links for publicly available datasets
- A description of any restrictions on data availability
- For clinical datasets or third party data, please ensure that the statement adheres to our policy

The authors declare that data that support this study are available within the article and its Supplementary Information files or available from the authors upon request.

Research involving human participants, their data, or biological material

Policy information about studies with human participants or human data. See also policy information about sex, gender (identity/presentation), and sexual orientation and race, ethnicity and racism.

Reporting on sex and gender	N/A
Reporting on race, ethnicity, or other socially relevant groupings	N/A
Population characteristics	N/A
Recruitment	N/A
Ethics oversight	N/A

Note that full information on the approval of the study protocol must also be provided in the manuscript.

Field-specific reporting

Please select the one below that is the best fit for your research. If you are not sure, read the appropriate sections before making your selection.

☒ Life sciences ☐ Behavioural & social sciences ☐ Ecological, evolutionary & environmental sciences

For a reference copy of the document with all sections, see nature.com/documents/nr-reporting-summary-flat.pdf

Life sciences study design

All studies must disclose on these points even when the disclosure is negative.

Sample size	Sample size for each experiment is indicated in the legends. No statistical methods were used to predetermine sample sizes. Sample sizes were determined by previous experiments in our laboratory in order to achieve 95% power for two-sided t-test .(PMID: 32541831, 37848037, 37848037, 26358190)
Data exclusions	No data were excluded from the analyses.
Replication	All experimental findings were reproduced in multiple independent experiments. For experiments using mouse peritoneal macrophages, each independent experiment used mouse peritoneal macrophages isolated from another mouse. For each figure, the number of independent experiments or biological replicates is indicated in the figure legends. Western blot pictures are from a representative experiment and the number of independent repeats is clearly indicated in the figure legends.
Randomization	For in vitro experiments, mouse peritoneal macrophages were isolated from randomly chosen wild-type or KO mice. For in vivo experiments, wild-type or KO mice were randomly allocated into experimental groups. All mice were age- and sex- matched. We also used both male and female mice for the experiments.
Blinding	The investigators were blinded during data collection and analysis.

Reporting for specific materials, systems and methods

We require information from authors about some types of materials, experimental systems and methods used in many studies. Here, indicate whether each material, system or method listed is relevant to your study. If you are not sure if a list item applies to your research, read the appropriate section before selecting a response.

Materials & experimental systems

n/a	Involved in the study	
☐	☒	Antibodies
☐	☒	Eukaryotic cell lines
☒	☐	Palaeontology and archaeology
☐	☒	Animals and other organisms
☒	☐	Clinical data
☒	☐	Dual use research of concern
☒	☐	Plants

Methods

n/a	Involved in the study	
☒	☐	ChIP-seq
☐	☒	Flow cytometry
☒	☐	MRI-based neuroimaging

Antibodies

Antibodies used

Anti-cGAS (D3O8O, 31659, 1:1000 for WB), anti-STING (D1V5L, 50494, 1:1000 for WB), anti-p-IRF3 (Ser396, 4947, 1:1000 for WB), anti-p-STAT1 (Tyr701, 9167, 1:1000 for WB), anti-TBK1 (3013, 1:1000 for WB), anti-IRF3 (4302, 1:1000 for WB) and anti-biotin (D5A7,5571, 1:1000 for WB) antibodies were purchased from Cell Signaling Technology.
Anti-p-TBK1 (ab109272, 1:1000 for WB) and anti-GM130 (ab52649, 1:200 for IF) antibodies were purchased from Abcam.
Anti-FLAG (F1804, 1:2000 for WB) antibody was purchased from Sigma-Aldrich.
Anti-β-actin (66009-I-IgF1804, 1:2000 for WB) antibody was purchased from Proteintech.
Anti-ADH5 (A13459, 1:1000 for WB) antibody was purchased from ABclonal.
Anti-hSTING (MAB7169,10μg/ml), used for immunofluorescence, was obtained from R&D Systems.
HRP-Goat Anti-Mouse IgG (SA00001-1,1:5000 for WB) and HRP-Goat Anti-Rabbit IgG (SA00001-2,1:5000 for WB) were purchased from Proteintech as secondary antibody.
Alexa Fluor 633 (A-21071, 1:500 for IF) and 488 (A-11059, 1:500 for IF) were purchased from Thermo Fisher Scientific as secondary antibody.

Validation

All antibodies were obtained from indicated commercial vendors with ensured quality. All the antibodies used in this study have been validated by the vendors as indicated on the websites. Citations are listed as below:
Anti-cGAS (Cell Signaling Technology, D3O8O, 31659) validate in mouse for WB/IP: Zheng Liu, et. al. XBP1 deficiency promotes hepatocyte pyroptosis by impairing mitophagy to activate mtDNA-cGAS-STING signaling in macrophages during acute liver injury. Redox Biol. 2022 Jun;52:102305.
Anti-STING (Cell Signaling Technology, D1V5L, 50494) validate in human/mouse/rat for WB/IP: Lili Tao, et. al. AKT1 Is Required for a Complete Palbociclib-Induced Senescence Phenotype in BRAF-V600E-Driven Human Melanoma. Cancers (Basel). 2022 Jan 23;14(3):572.
Anti-p-IRF3 (Cell Signaling Technology, Ser396, 4947) validate in human/mouse for WB: Ee Shan Pang, et. al. Discordance in STING-Induced Activation and Cell Death Between Mouse and Human Dendritic Cell Populations. Front Immunol. 2022 Feb 25;13:794776.
Anti-p-STAT1 (Cell Signaling Technology, Tyr701, 9167) validate in human/mouse for WB/IP: Xiaoxiao Zhu, et. al. MiR-19a-3p Suppresses M1 Macrophage Polarization by Inhibiting STAT1/IRF1 Pathway. Front Pharmacol. 2021 May 4;12:614044.
Anti-TBK1 (Cell Signaling Technology, 3013) validate in human/mouse for WB:Wang M, et. al. Inhibition of tumor intrinsic BANF1 activates antitumor immune responses via cGAS-STING and enhances the efficacy of PD-1 blockade. J Immunother Cancer. 2023 Aug;11(8):e007035.
Anti-IRF3 (Cell Signaling Technology, 4302) validate in human/mouse for WB:Liu J,, et. al. Medicinal plant-derived mtDNA via nanovesicles induces the cGAS-STING pathway to remold tumor-associated macrophages for tumor regression. J Nanobiotechnology. 2023 Mar 6;21(1):78.
Anti-biotin (D5A7,5571, 1:1000 for WB) recognizes biotin attached to proteins:Yang J, et. al.. TAK1 Improves Cognitive Function via Suppressing RIPK1-Driven Neuronal Apoptosis and Necroptosis in Rats with Chronic Hypertension. Aging Dis. 2023 Oct1;14(5): 1799 -1817.
Anti-p-TBK1 (Abcam, ab109272) validate in mouse/human for WB: Banerjee A et. al. Experimental and natural evidence of SARS-CoV-2-infection-induced activation of type I interferon responses. iScience 24:102477 (2021).
Anti-GM130 (Abcam, ab52649) validate in human for WB/IF: Peijun Li et. al. Doublecortin facilitates the elongation of the somatic Golgi apparatus into proximal dendrites. Mol Biol Cell. 2021 Mar 1;32(5):422-434.
Anti-Flag(Sigma-Aldrich, F1804) validate in human for WB/IP: Huifang Hu et. al. ZKSCAN3 counteracts cellular senescence by stabilizing heterochromatin. Nucleic Acids Research.48(11):6001-6018 (2020).
Anti-β-actin (Proteintech, 66009-I-Ig) validate in human/mouse for WB/IP: Lorenzo DN et. al. Cell-autonomous adiposity through increased cell surface GLUT4 due to ankyrin-B deficiency. Proc Natl Acad Sci U S A. 2017 Nov 28;114(48):12743-12748.
Anti-ADH5 (ABclonal, A13459) validate in human/mouse for WB.
Anti-hSTING (R&D Systems, MAB7169) validate in human for WB/IF: Xiang Gui et al. Autophagy induction via STING trafficking is a primordial function of the cGAS pathway. Nature. 2019 Mar;567(7747):262-266.

Eukaryotic cell lines

Policy information about cell lines and Sex and Gender in Research

Cell line source(s)

HEK293T and THP-1 cells were purchased from the American Type Culture Collection. 293-Dual hSTING-A162 cells were obtained from InvivoGen. BJ cells were purchased from Shanghai Fuheng Biotechnology Co., Ltd.

Authentication

None of the cell lines have been authenticated.

Mycoplasma contamination

The cell lines were not tested for mycoplasma contamination.

Commonly misidentified lines (See ICLAC register)	No commonly misidentified cell lines were used.

Animals and other research organisms

Policy information about studies involving animals; ARRIVE guidelines recommended for reporting animal research, and Sex and Gender in Research

Laboratory animals	Adh5-deficient (S-KO-00928) mice were generated by Cyagen Biosciences using CRISPR/Cas9-mediated genome editing. Sting1-deficient mice were obtained from Jackson Laboratory. C57BL/6 mice were from Vital River Laboratory Animal Technology Co. (Beijing, China). Mouse primary peritoneal macrophages were obtained from 6-11 weeks old male or female mice. For in vivo experiments, 7 weeks old females were used. All mice were housed in a pathogen-free facility at the Model Animal Research Center of Shandong University. They were kept in day/night cycles (12h each), with temperature of 20–26°C and humidity 40–70%.
Wild animals	The study did not involve wild animals.
Reporting on sex	Mouse primary peritoneal macrophages were obtained from male or female mice. For in vivo experiments, female mice were used.
Field-collected samples	The study did not involve samples collected from field.
Ethics oversight	All animal experiments were performed in compliance with the National Institutes of Health Guide for the Care and Use of Laboratory Animals with approval from the Scientific Investigation Board of the School of Basic Medical Science, Shandong University, Jinan, Shandong Province, China.

Note that full information on the approval of the study protocol must also be provided in the manuscript.

Plants

Seed stocks	N/A
Novel plant genotypes	N/A
Authentication	N/A

Flow Cytometry

Plots

Confirm that:

☐ The axis labels state the marker and fluorochrome used (e.g. CD4-FITC).

☐ The axis scales are clearly visible. Include numbers along axes only for bottom left plot of group (a 'group' is an analysis of identical markers).

☐ All plots are contour plots with outliers or pseudocolor plots.

☒ A numerical value for number of cells or percentage (with statistics) is provided.

Methodology

Sample preparation	The production of ROS in cells was measured using the H2DCFDA probe (S9687, Selleck, USA) according to the manufacturer's recommendation.
Instrument	CytoFLEX cytometer instrument (Beckman Coulter, Brea, CA, USA)
Software	FlowJo v10.8 software
Cell population abundance	After removing dead and adherent cells, approximately 70% of the PMs are left for analysis.
Gating strategy	Dead and adherent cells were discriminated out by gating on live/dead population.

☐ Tick this box to confirm that a figure exemplifying the gating strategy is provided in the Supplementary Information.
